# Age- and Gender Dependent Liver Fat Content in a Healthy Normal BMI Population as Quantified by Fat-Water Separating DIXON MR Imaging

**DOI:** 10.1371/journal.pone.0141691

**Published:** 2015-11-10

**Authors:** Erika J. Ulbrich, Michael A. Fischer, Andrei Manoliu, Magda Marcon, Roger Luechinger, Daniel Nanz, Caecilia S. Reiner

**Affiliations:** 1 Institute for Diagnostic and Interventional Radiology, University Hospital Zurich, Zurich, Switzerland; 2 Institute for Biomedical Engineering, University and ETH Zurich, Zurich, Switzerland; Northwestern University Feinberg School of Medicine, UNITED STATES

## Abstract

**Objectives:**

To establish age- and sex-dependent values of magnetic resonance (MR) liver fat-signal fraction (FSF) in healthy volunteers with normal body-mass index (BMI).

**Methods:**

2-point mDIXON sequences (repetition time/echo time, 4.2msec/1.2msec, 3.1msec) at 3.0 Tesla MR were acquired in 80 healthy volunteers with normal BMI (18.2 to 25.7 kg/m2) between 20 and 62 years (10 men/10 women per decade). FSF was measured in 5 liver segments (segment II, III, VI, VII, VIII) based on mean signal intensities in regions of interest placed on mDIXON-based water and fat images. Multivariate general linear models were used to test for significant differences between BMI-corrected FSF among age subgroups. Pearson and Spearman correlations between FSF and several body measures were calculated.

**Results:**

Mean FSF (%) ± standard deviations significantly differed between women (3.91 ± 1.10) and men (4.69 ± 1.38) and varied with age for women/men (p-value: 0.002/0.027): 3.05 ± 0.49/3.74 ± 0.60 (age group 20–29), 3.75 ± 0.66/4.99 ± 1.30 (30–39), 4.76 ± 1.16/5.25 ± 1.97 (40–49) and 4.09 ± 1.26/4.79 ± 0.93 (50–62). FSF differences among age subgroups were significant for women only (p = 0.003).

**Conclusions:**

MR-based liver fat content is higher in men and peaks in the fifth decade for both genders.

## Introduction

The liver plays a central role in lipid metabolism. About 80% of the liver steatosis cases are due to alcohol [1] while metabolic causes include metabolic syndromes, insulin resistance, nutrition, obesity, medication, and inflammatory processes [2]. Liver steatosis is a risk factor for type II diabetes and cardiovascular disease [3], for the development of liver cirrhosis [4] and hepatocellular carcinoma (HCC) [5] as well as for the development of postoperative complications following liver surgery [6].

Traditionally, biopsy in combination with histopathological analysis is the gold standard for liver fat quantification. In the meanwhile there are several magnetic resonance (MR) imaging techniques available for noninvasive liver fat detection: chemical shift imaging [7–11], frequency-selective imaging [12–14] and MR spectroscopy [15]. Regardless of the MR imaging technique used, the key step for liver fat quantification is to separate the fat- and water-signal contributions of the net MR signal.

The two-point DIXON technique is a technique that can rapidly (within a single breath hold) produce highly resolved separated water-only and fat-only images of extended anatomical volumes [16], e.g., of the liver. DIXON-based liver fat quantification was shown to be of higher accuracy than standard histopathological assessment of liver fat and is therefore increasingly used for liver fat quantification today [17].

Knowledge of the normative MRI-based hepatic fat content of a given age group might allow screening for liver steatosis and help the clinician to better estimate the risk of systemic disease, liver cirrhosis and postoperative complications. Therefore, we prospectively quantified liver fat content in healthy volunteers, assessed as MRI fat signal fraction by two-point DIXON-fat-water-separation MR imaging at 3.0 Tesla.

The aim of this work was to define age- and sex-dependent reference standards of liver-fat-fractions.

## Material and Methods

### Study subjects and Clinical Examination

This was a prospective single-center study with institutional review-board approval from the cantonal ethics committee (number KEK: 2010–0437) and written informed consent from all study subjects. The study was Health Insurance Portability and Accountability Act (HIPAA) compliant and none of the authors had a financial interest. The present study included study subjects of a larger clinical trial of whole-body MR imaging of healthy volunteers examined between 2011 and 2014 (unpublished data).

A total of 80 healthy volunteers were consecutively imaged with whole-body MRI for a larger clinical trial (40 women; mean age, 39.60 ±12.16 years; age range, 21–62 years; 40 men; mean age, 39.70 ± 11.23 years; age range, 20–61 years; 10 men/10 women per decade) and included in the current study of liver-fat assessment. One volunteer in each gender group was slightly older than 60 years (f, 62 years; m, 61 years).

Inclusion criteria were *(a)* normal BMI (18.2 to 25.7 kg/m2) [18]; *(b)* age between 20 and 62 years*; (c)* healthy. Exclusion criteria were: *(a)* contraindication for MR imaging (claustrophobia, metal fragments and implants, stents, pacemaker, pregnancy); *(b)* surgery, especially osteosynthesis because of the susceptibility artefacts and associated severe fat/water signal swaps; *(c)* systemic diseases (chronic obstructive pulmonary diseases, diabetes, metabolic diseases, rheumatologic disorders, tumors, chronic pain syndrome); (d) vascular problems (coronary heart disease, peripheral artery disease); (e) alcohol addiction, drug abuse. All subjects had to fill in a questionnaire concerning the above mentioned inclusion and exclusion criteria.

For each subject the following parameters were determined: age, height, weight, BMI, waist and hip circumference and abdominal girth. The body fat was measured with a bioelectrical impedance analyzer (BIA) via the electrical body resistance using foot sensor pads on a bathroom-scale like device (TANITA UM-018, Tanita Corp, Arlingthon Heights, Ill).

All subjects were screened using ‘(National) Olympics First Sports Medicine Interview’ health survey, including comprehensive questions determining medical history, exercise, weight change (Y/N), smoker (Y/N), alcohol (Y/N) and drug use (Y/N).

### Data acquisition and Data Analysis

All MR data were acquired on a 3.0 Tesla MR unit (Ingenia, Philips Healthcare, Best, The Netherlands). The subjects were positioned supine with both arms along the body. A 16 channel posterior coil, which was integrated in the table and automatically centered at the imaged anatomy, and two 16 channel anterior coils were used for signal reception. The scanner’s dual transmit body coil was used for radiofrequency transmission. For each volume a B1 map was acquired and used to optimize RF transition.

The study MR imaging protocol included axial mDIXON-sequences (2-point DIXON fat-water separation) of the whole body. The mDIXON sequence had the following parameters: sequence type, 3D FFE T1; number of echoes, 2; acquired voxel dimensions (mm), 2.0, 2.0, 4.0; reconstructed voxel dimensions (mm), 1.0, 1.0, 2.0; inter-slice gap (mm), 0.0; field of view, 560 x 352; number of sections, 80; TR, 4.2 msec; TE, 1.2 and 3.1.msec; flip angle, 5°; number of signal averages, 2; SENSE acceleration factor (AP/SI), 2.0 and 2.0; fold-over direction, AP; water-fat shift (pixel), 0.292; receiver bandwidth (Hz pixel^-1^), 1485.1; single series acquisition time per sequence block (sec), 16.4. For this study only the two relevant axial mDIXON sequence blocks containing the parts of the liver were used for the current read-out.

**Fig 1:**
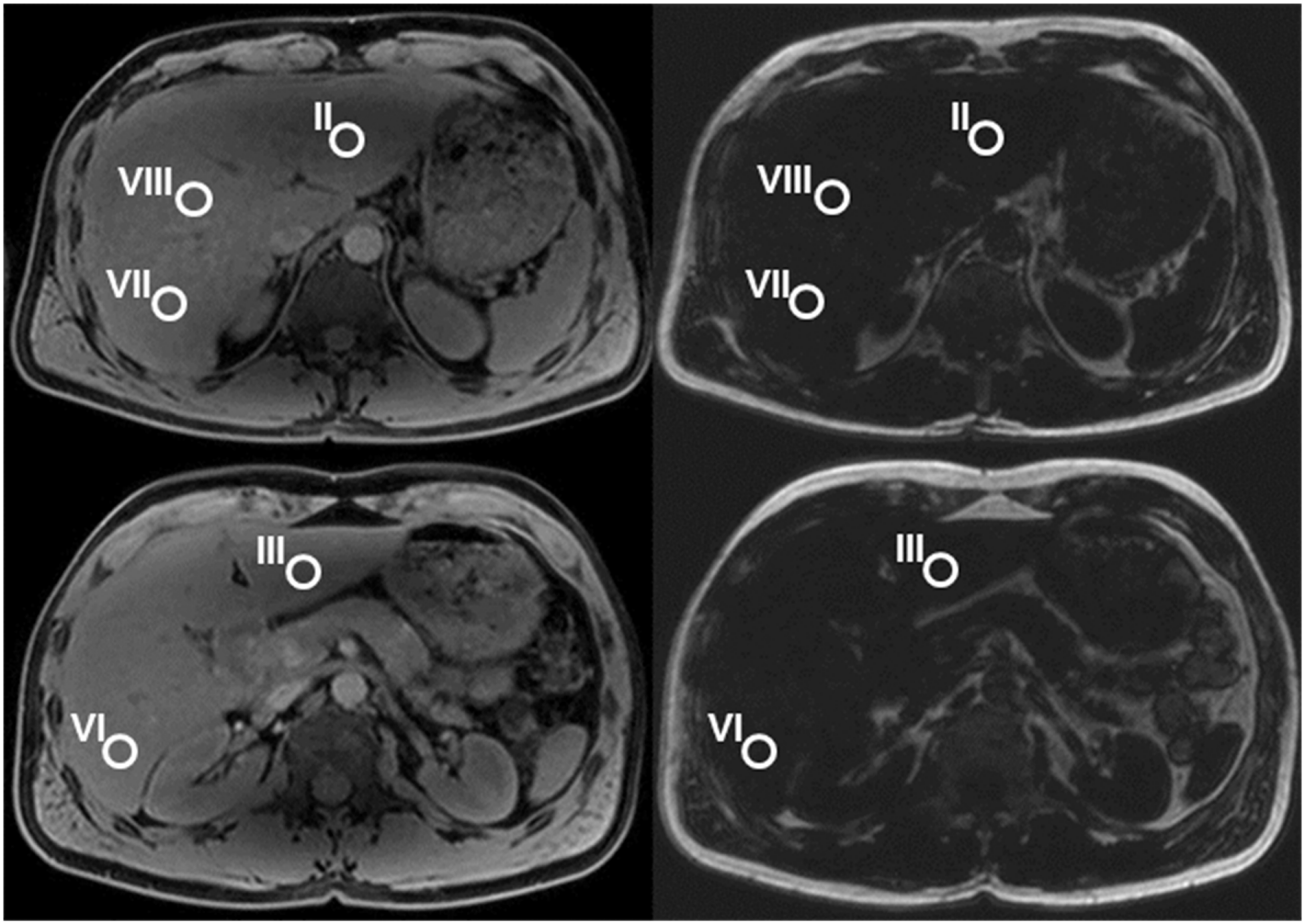
Axial water-signal-only (left side) and fat-signal-only (right side) MR images in a 31-year-old woman. Regions of interest (ROIs) were placed in liver segments II, III, VI, VII and VIII on the water-signal-only images and copied to the fat-signal-only images.

Images were analyzed with the DICOM Viewer Osirix v 5.9 (PIXMEO^®^, Geneva, Switzerland) by two board-certified radiologists, one specialized in liver MR imaging with 9 years of experience (CSR) and one with 12 years of experience in Radiology (EJU). Mean dual-echo fat-signal fractions (FSF) of the liver were determined in 5 liver segments: liver segment II (left lobe, superior portion, medial to the left liver vein), III (left lobe, inferior portion, medial to the falciform ligament), VI (right lobe, posteroinferior to the portal vein), VII (right lobe, posterosuperior, dorsal to the right liver vein), VIII (right lobe, anterosuperior, between right and middle liver vein). In each of these 5 liver segments a round region of interest (ROI) with a diameter of 1.5 cm was placed to measure signal intensities. The ROIs were drawn on water signal-only images including liver parenchyma only and avoiding vessels, biliary ducts or areas affected by imaging artefacts, and were then copied to the fat signal-only images (Fig 1).

The mean water (signal _water_) and fat (signal _fat_) signal intensities in the respective ROIs were used to calculate the fat signal fraction, FSF, according to the following equation [19]:

$$\text{FSF }\left[\%\right]\;=\;\left(\text{signa}{\text{l}}_{\text{fat}}/\;\left(\text{signa}{\text{l}}_{\text{water}}+\text{ signa}{\text{l}}_{\text{fat}}\right)\right)\text{ x }100$$

For further analysis the mean value of FSF of the 5 ROIs (= FSFtot) was calculated.

### Statistical Analysis

Descriptive statistics of the measured parameters were obtained (reported as mean ± standard deviation) and their distributions tested for normality with the Kolmogorov-Smirnov test.

From a subset of the study cohort (20%; 2 subjects per decade per gender) an intra-class correlation coefficient was calculated to evaluate the interreader agreement—according to Kundel and Polansky [20] an ICC of 0.21–0.40 indicated poor, 0.41–0.60 moderate, 0.61–0.80 good and ≥ 0.81 an excellent agreement (ICC = 1.00, “perfect” agreement).

ANOVA analysis was performed to assess potential FSF differences among age subgroups, gender and to each other. In addition, multivariate analysis was performed with FSF as dependent value and BMI as covariate of no interest to account for the potential influence of BMI on liver fat in different age subgroups.

Correlations between MR FSF measurements and age, weight, height, BMI, waist-, hip-, abdominal girth-measurements, waist-to-hip ratio (WHR) and the body fat measured by the BIA, were assessed by Pearson correlation analysis.

Correlations between FSF and the following parameters were assessed with the Spearman rank correlation: age group, weight change, diet, nicotine intake, and alcohol intake. Due to Bonferroni-correction for multiple comparisons (n = 5) p-values <0.01 were deemed statistically significant.

All statistical analyses were performed using commercially available software (SPSS, release 22.0, Chicago, IL USA).

## Results

Mean values, standard deviations, and ranges for BMI, WHR, waist-, abdominal- and hip-girth measurements, and body fat percentages from impedance measurements are shown in Table 1.

**Table 1:** Gender specific descriptive body characteristics of the study-group individuals.

Parameter	Women (n = 40)	Men (n = 40)	p-value (t-test)
**BMI / kg m** ^**-2**^	21.7 ± 2.1 (18.2–25.7)	23.0 ± 1.8 (19.4–25.6)	0.005
**Waist girth / cm**	71.4 ± 5.6 (61–85)	84.1 ± 6.1 (75–99)	< 0.001
**Abdominal girth / cm**	77.8 ± 7.8 (66–97)	89.0 ± 6.1 (78–102)	< 0.001
**Hip girth / cm**	93.8 ± 6.9 (78–109)	99.4 ± 11.4 (83–160)	0.010
**WHR**	0.8 ± 0.1 (0.7–1)	0.9 ± 0.1 (0.5–1)	< 0.001
**Body Fat BIA / %**	28.0 ± 5.6 (13–39)	17.8 ± 4.7 (5.4–31)	< 0.001

Abbreviations: WHR = waist-to-hip ratio, BMI = body mass index, BIA = body fat measured via fat analyzer

**Fig 2:**
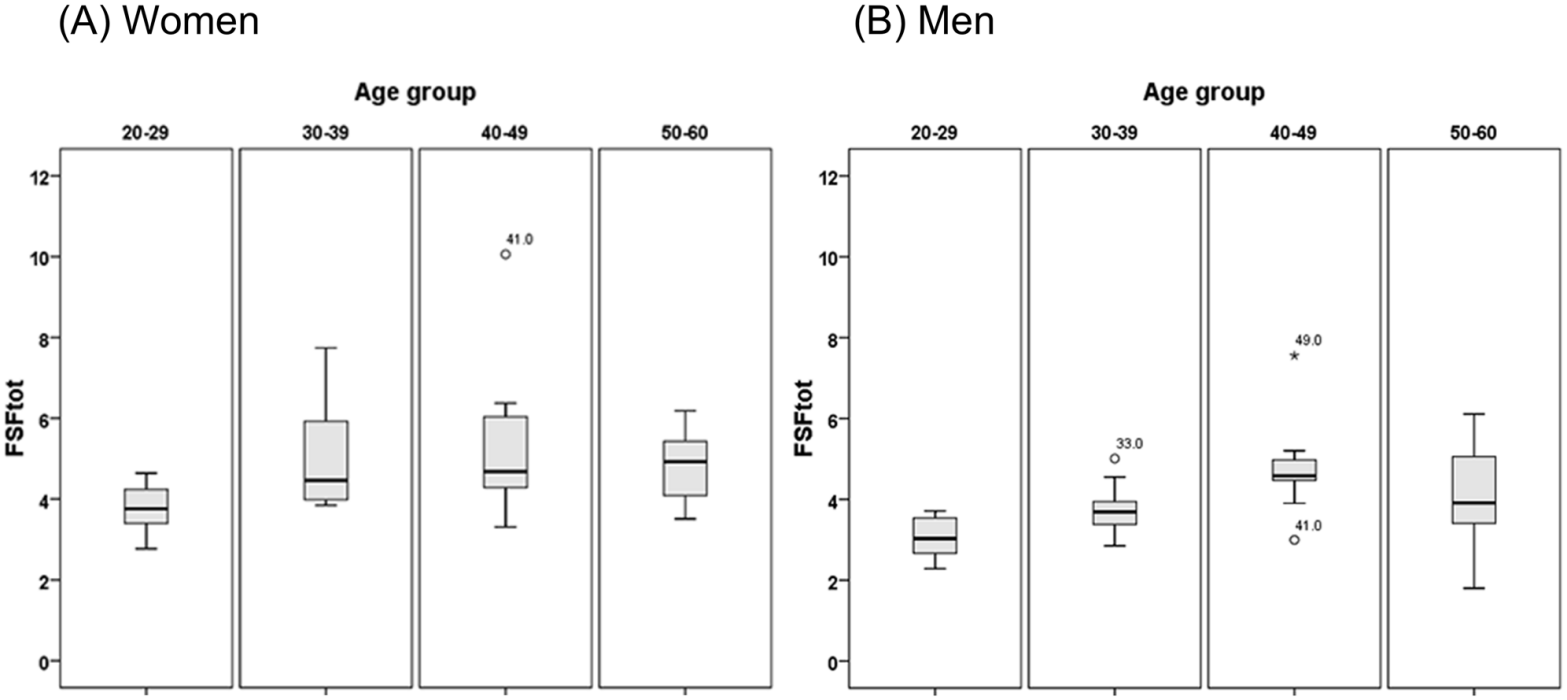
Age group dependence of mean FSFtot in Women (Fig 2a) and Men (Fig 2b) demonstrated in Box-and-Whisker plots. The line within the box represents the median value. Boxes represent 25^th^ to 75^th^ percentiles. The lines outside indicate the 10^th^ and 90^th^ percentiles.

**Fig 3:**
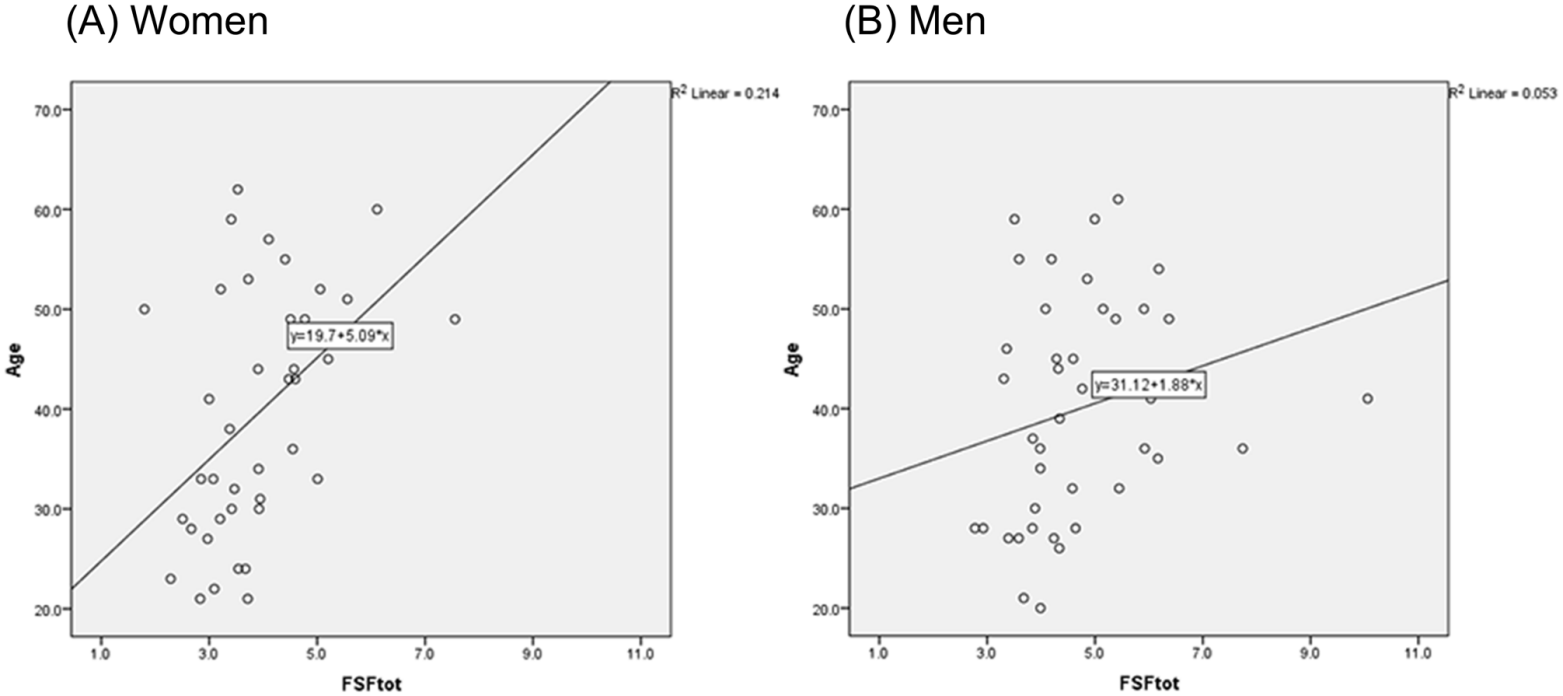
Scatter plots with appropriate fitting lines to present the FSF values in relation to age (Fig 3a: women, Fig 3b: men).

Age-group specific mean liver FSFs (%) of women and men are shown in Table 2 and graphically summarized in Figs 2 and 3. For both genders, FSFtot increased with age and peaked in the age group 40–49 years, followed by a decrease in the older groups. Within the whole age span of 20–62 years, FSFtot for women ranged from 1.80 to 7.56% (mean 3.91% ± 1.10), FSFtot for men ranged from 2.77 to 10.06% (mean 4.69% ± 1.38). FSFtot was significantly different in women and men (F-value = 7.809; p = 0.007). Comparing FSFs in the different segments, averaged over all age groups (20–62), the largest FSF values were measured in liver segment VI in both genders (4.2±1.4% in women and 5.1±1.6% in men), but without the difference reaching statistical significance, therefore suggesting only a trend for segment VI having highest fat content.

**Table 2:** Age-group and gender-specific mean FSF values. Bold numbers indicate the highest FSF values.

Age [years]	Mean FSF±SD / %	
	Women	Men
**20–29**	3.05±0.49	3.74±0.60
**30–39**	3.75±0.66	4.99±1.30
**40–49**	**4.76±1.16**	**5.25±1.97**
**50–62**	4.09±1.26	4.79±0.93
**20–62**	3.91±1.10	4.69±1.38

### Interreader agreement of FSF

The interreader agreement between the two readers was excellent for both gender with an ICC 0.894 for women and 0.972 for men.

### Correlation of FSF with age and age subgroups:

Spearman and Pearson correlations of FSFtot with age group and with age were significant for women (p = 0.002 and 0.003), but not for men (p = 0.027, 0.153) (see Table 3).

**Table 3:** Pearson coefficients (R-value) for correlations between mean FSFs and descriptive body parameters (age, height, weight-, waist-, hip-, abdominal-girth measurements, WHR, BMI, BIA) and Spearman coefficients (R-value) for correlations between mean FSFs and age group. Italics indicate statistical significance. P-values < 0.05 (before Bonferroni correction, correction by factor 5) were considered statistically significant.

Parameter	Coefficient	FSF	
		Women	Men
**age group**	r-value	0.478	0.349
	p-value	0.002	0.027
**age**	r-value	0.462	0.23
	p-value	0.003	0.153
**Height**	r-value	0.097	0.033
	p-value	0.552	0.838
**Weight**	r-value	0.127	0.221
	p-value	0.434	0.17
**Waist G**	r-value	0.157	0.38
	p-value	0.334	0.016
**Abd. G**	r-value	0.076	0.368
	p-value	0.639	0.019
**Hip G**	r-value	0.04	-0.039
	p-value	0.806	0.809
**WHR**	r-value	0.051	0.307
	p-value	0.753	0.054
**BMI**	r-value	0.12	0.235
	p-value	0.461	0.145
**BIA**	r-value	0.12	0.5
	p-value	0.472	0.001

Abbreviations: Waist G, Abd. G, Hip G = waist, abdominal or hip girth, respectively, WHR = waist-to-hip ratio, BMI = body mass index, BIA = body fat measured with a bioelectrical impedance analyzer

The FSF difference among age subgroups for women was significant for FSFtot (p = 0.003; see Table 4), but not significant after correction for BMI in a multivariate analysis.

**Table 4:** ANOVA analysis and multivariate analysis (corrected for BMI) of differences of mean FSF values among age groups in both genders (F-value = ratio of mean square values). Italics indicate statistical significance. P-values < 0.05 (before Bonferroni correction, correction by factor 5) were considered statistically significant.

Differences among age groups		FSF	
		Women	Men
**ANOVA**	F-value	0.561	2.589
	p-value	0.003	0.068
**Corrected**	F-value	1.22	0.708
**for BMI**	p-value	0.277	0.406

There was no FSF difference among age subgroups for men for FSFtot (p = 0.068; see Table 4) with similar results after correction for BMI in the multivariate analysis.

### Correlation of FSF with Body parameters:

There were no significant Pearson correlations of FSFtot with height, weight, BMI, waist-, hip- and abdominal girth-measurements, WHR and body fat measurements by BIA in women (see Table 3).

In men, there was significant Pearson correlation only between FSFtot and body fat measurements by BIA (p = 0.001) and a tendency towards significant correlations between FSFtot with waist and abdominal girth (p = 0.016 and 0.019; see Table 3).

### Correlation of FSF with weight change and diet in the last two years, nicotine and alcohol intake

3 women and 2 men declared slight weight changes, 3 women and 1 man declared to be on a diet, 7 women and 7 men were smokers and 11 women and 16 men declared a regular consumption of alcohol in low quantities.

The Spearman correlations between FSFtot and these lifestyle parameters were not significant.

## Discussion

We report two-point mDIXON fat-signal fractions as a measure of liver fat content in a large cohort of healthy volunteers of varying age (20–62 years) and gender. Such data are of critical importance for potential future DIXON-based liver fat quantification in clinical routine, which needs to distinguish normal from stratified elevated liver-fat content to allow a risk assessment in patients in suspicion of fatty liver disease. A direct conversion of MR imaging derived fat-signal fractions to actual fat content, e.g., in milligram fat per gram liver tissue, is the subject of a large on-going effort in the MR imaging community, which will require a considerable amount of—potentially disease-specific—validation, far beyond the scope of the current study.

Up to now, liver-fat content is most often determined by liver biopsy in combination with histopathological analysis. Due to their invasiveness, biopsies are seldom performed in healthy individuals without a clinical indication and to date no cross-sectional overview of histology-based analysis of liver fat content in a healthy living population has been acquired. Visual biopsy evaluation typically relies on cell counts and results in higher apparent fat-content estimations than volumetric measurements. Evaluation with digital image analysis and computer-assisted morphometry as well as DIXON-based MR imaging typically estimate fat contents that are smaller by a factor of two compared to cell-counting based evaluations [21, 22]. Traditionally, liver fat contents above 50 mg/g (5% by wet weight) are considered diagnostic of hepatic steatosis [23].

Noninvasive methods like localized proton magnetic resonance spectroscopy (MRS) have the potential to accurately measure hepatic triglyceride content [15, 24, 25]. However, in case of non-homogeneous liver tissue, MRS is subject to similar sampling errors like biopsy. Nowadays MR imaging has been promoted as a new gold standard for liver fat quantification as it can measure intrahepatic fat with high accuracy [17, 26]. In contrast to biopsies, this method is non-invasive and offers a representative assessment of the whole liver.

In a larger trial of 2,287 participants, hepatic triglyceride positively correlated with BMI. 95% of the 345 participants with normal BMI and no risk factors for fatty liver disease had less than 5.56% hepatic triglyceride by weight, with a minimum of 1.9% of tissue by weight [2, 27], as measured by MRS. These values seem consistent with our FSF results in healthy volunteers with mean liver FSF values of 3.91% ± 1.10 in women and 4.69% ± 1.38) in men. Observed discrepancies in the maximum range of values might be due to the differences in study subjects and are most probably age related.

Studies in patients with chronic hepatitis C biopsied before therapeutic trials showed correlation of liver steatosis with BMI [28]. In contrast, in patients with morbid obesity (liver biopsied within laparoscopic obesity surgery), the degree of liver steatosis did not correlate significantly with the BMI [29]. Hines et al. [30] reported an exponential relationship between MR fat-signal fraction and BMI using a three-dimensional spoiled gradient echo sequence reconstructed with a T2* corrected IDEAL algorithm (with single peak and with a low flip angle of 5°). In our study we did not find a significant correlation of liver fat signal fraction with BMI, but our healthy population ranged within the normal BMI range only. Moreover, in our study, body fat measurements by BIA correlated only with liver FSFs in men.

According to NIH/WHO BMI guidelines as reported by Gallagher et al [31] international healthy body fat percentages (measured via dual-energy X-ray absorptiometry) increase with age. Although there was no significant correlation between liver FSFs and BMI and body fat (measured with impedance scales) in our study, liver FSFs showed a significant correlation with age for women and a tendency for men indicating that liver fat content increases with age similar to body fat. Interestingly, the FSFs of our study peaked in the fifth decade for both gender (statistically significant for women only) and decreased again thereafter. This is contradictory to the general assumption of steady increase of liver steatosis with aging [32].

In the last years, liver fat was quantified within multiple DIXON-based MR studies. These studies consistently found a strong correlation between MR imaging-based and MR spectroscopy based fat-signal fractions [33, 34] as well as with histopathologic examination [10, 17, 22]. The DIXON sequence and post processing capitalize on the fact that transverse magnetization of fat and water protons precess at different frequencies (chemical shift) resulting in an echo-time dependent phase shift that allows separation of the signal components, the computation of water-only and fat-only images [16] and, correspondingly of fat signal fraction (FSF) [19]. Typically, an acquisition of at least 2 echoes is required for imaging-based fat-water separation, with more echoes allowing an improved correction of interfering effects, such as T1 bias, due to longer TR, B0-field distortion, or T2*-shortening caused by increased tissue-iron content and concomitant rapid decay of transverse magnetization [35]. In a recent study [22] at 1.5 Tesla, with a dual-echo spoiled gradient-echo sequence (LAVA-FLEX) with TR, 6.9 msec; TE, 2.4, 4.8 msec; flip angle 13°, in patients not suffering from underlying liver disease, such as liver cirrhosis and hemosiderosis, the mean FSF ranged from 3 to 26% (mean 8.3% ± 6.2) with correlating histopathologic hepatosteatosis of <5 to 90% (mean 28% ± 28%). In another 1.5 Tesla-study [36] (dual-echo spoiled gradient-echo sequence (LAVA-FLEX); TR, 6.3–6.7 msec; TE, 3.1–3.2, 6.4–6.5 msec; flip angle 12°) in patients without diffuse liver disease, the FSFs in liver segment II and VIII ranged from 1.0 to 33.9% (mean 9.6% ± 5.2) and from 1.6 to 32.7% (mean 9.3% ± 5.1) respectively. Hines et al [37] (1.5 T, 6-echo spoiled gradient-echo sequence (IDEAL); TR, 13.7 msec; first TE, 1.3 msec; echo spacing, 2.0 msec; flip angle 5°) measured similar FSFs in MR in a mixed patient cohort with a range of 0 to 23% (mean 5.72% ± 6.03) for women and a range of 0.54–36.45% (mean 5.71 ± 9.00%) for men. In our study (3 Tesla; dual-gradient echo spoiled gradient-echo sequence (mDIXON); TR, 4.2 msec; TE, 1.2, 3.1 msec; flip angle 5°), all individuals (with normal BMI) showed a liver fat signal fraction (FSF) range of 1.80 to 7.56% (mean 3.91% ± 1.10) for women and a range of 2.77 to 10.06% (mean 4.69% ± 1.38) in men. As we selected strictly for healthy volunteers in this study, the FSF of our study cohort is lower than in the recent studies with patients [22, 36] even though differences in imaging parameters and field strength have to be taken into account for lower FSFs.

The following study limitations should be noted. In areas of extensive breathing artifacts, especially at the liver dome, no measurements were made. Occasionally, fat-/water swaps occurred, especially in the liver dome (segment VII/VIII), but no ROIs were drawn in these regions for the purpose of the current study. We used a dual-echo sequence for fat quantification which is prone to potentially confounding T1 and T2* relaxation effects [38, 39], but a recent paper [40] showed no difference in dual fat fraction and triple fat fraction in healthy children with a mean value of 2.3±2.0% on dual-echo MRI and 2.9±1.4% on triple-echo MRI. Dual-echo imaging was shown to measure lower FSF values in patients with pathological liver iron deposition [41], an effect, which in part may be corrected with multi-echo imaging [42]. In images acquired with as short echo times relative to the T2* of healthy livers as in our study, it is normally assumed that FSF distortion by interference from T2* decay is negligible [10, 34, 41]. To minimize potential iron interference in our healthy population we excluded pathological liver iron deposition visually on the correlated T1-weighted images (exclusion of signal intensity drop, n = 0/80). To minimize T1 bias [38, 39] the excitation flip angle was lowered to 5°, which is quite effective in approximating proton density-weighting of the image contrast with reasonably low T1 influence. The proton spectrum of physiological hepatic fat, as opposed to that of water, is further known to consist of a multitude of spectral lines [43], potentially also depending on an individually varying and/or disease-specific chemical composition. For fat-water separating liver imaging, the spectrum of hepatic triglycerides is typically modeled to consist of 6 or 7 resolved peaks with the largest peak of mostly methylene protons accounting for ca. 70% of the total signal [43]. An independent modeling of the amplitude of the different peaks in fat-water separating reconstruction requires the acquisition of multiple echoes, which may also allow a correction for T2* decay and multi-peak fat spectrum modelling [42, 44]. Our dual-echo based imaging (two-point mDIXON) had an underlying fat-signal model with a single peak at 1.3 ppm, neglecting the smaller signals, and resulting in a slight underestimation of fat signal fraction, compare, e.g. the study of Hamilton et al [43]. However, since the dual-echo based approach has been proven quite successful in several studies [17, 22, 36] we decided to acquire only two signal echoes per excitation pulse, which allowed us to keep the repetition time short and to realize a tolerable total data-acquisition time at high spatial resolution.

An advantage of a whole body MRI protocol is the possibility to simultaneously quantify the fat-signal fraction in multiple organs, e.g. beyond the liver also in in pancreas, heart, kidneys, or muscles, in addition to allowing the calculation of indices such as total adipose tissue, visceral adipose tissue and abdominal subcutaneous adipose tissue. Thus, such a MR protocol may give a fast overview of the general health status of a patient, which might be valuable in the risk assessment of patients with e.g. coronary heart disease, metabolic syndroms (like diabetes), with a tumor history or sarcopenia

In conclusion, **DIXON-based liver fat content is higher in men than in women and peaks in the fifth decade for both genders.**

The knowledge of the normative hepatic fat content should help the clinician to better estimate the risk of long-term development of diabetes, cardiovascular disease, cancer and other disabling conditions.

## Abbreviations

ANOVA: analysis of variance
BMI: body mass index
BIA: body fat measured by a bathroom-scale like bioelectrical impedance analyzer (TANITA UM-018), body-fat estimation in weight percentage
FSF: fat signal fraction
FSFtot: mean value of all five FSFs
ROI: region of interest
SD: standard deviation
TE: echo time
TR: repetition time
WHR: waist-to-hip ratio
